# Effect of ethanol extract of boiled breadfruit (*Treculia Africana*) seed on the oral glucose tolerance, lipid profile, and body weight of normoglycemic albino rats

**DOI:** 10.1002/fsn3.626

**Published:** 2018-03-25

**Authors:** Kate Eleazu, Patric Maduabuchi, Chinedum Eleazu

**Affiliations:** ^1^ Ebonyi State University Abakaliki Nigeria; ^2^ Federal University, Ndufu‐Alike, Ikwo Ebonyi State Nigeria

**Keywords:** breadfruit, extract administration, lipid profile, oral glucose tolerance

## Abstract

The effect of ethanol extracts of boiled *Treculia africana* seed on the oral glucose tolerance (OGTT), lipid profile, and body weight of normoglycemic albino rats was determined. Fifteen male albino rats were used and were divided into three groups of five rats each. Groups 1 and 2 received 100 and 200 mg/kg of the extract, while group 3 (control group) received 1 ml/kg of normal saline. The experiment lasted for 28 days. The body weights of the rats were determined daily. OGTT was determined at week zero (before extract administration) and at weeks 2 and 4, respectively, following extract administration. Glycemic index (GI) of the extracts was calculated from the incremental area under the OGTT curve. The total cholesterol, high‐density lipoprotein (HDL) cholesterol, low‐density lipoprotein (LDL) cholesterol, and very‐low‐density lipoprotein (VLDL) cholesterol levels in the sera of the rats were determined using standard techniques. Atherogenic index (AI) and coronary risk index (CRI) of the rats were expressed as ratios of LDL cholesterol to HDL cholesterol and total cholesterol to HDL cholesterol, respectively. Following 2 weeks of the extract administration, the blood glucose for groups 1 and 2 rats declined to values ≤100 mg/dl after oral glucose loading. GI for the standard rat feeds and the extracts at 100 and 200 mg/kg by the second week of experimentation were 100, 114, and 96.09, respectively. GI for the extract at 100 mg/kg decreased to 103.63 at the 4th week, while that for the extract at 200 mg/kg increased to 98.07. The extract at 100 mg/kg increased the LDL cholesterol, AI, and CRI of the rats, suggesting that consumption of boiled African breadfruit may expose an individual to the risk of development of cardiovascular diseases. Finally, the study suggested that consumption of *T. africana* seed by a nondiabetic subject may have no effect on the glucose tolerance of the individual, while it will negatively impact on the glycemic status of a diabetic subject.

## 1 INTRODUCTION

Uncontrolled increase in postprandial glucose concentration is associated with greater risks of developing metabolic disorders, typically diabetes mellitus. It is well known that diabetes mellitus is one of the risk factors for atherosclerosis and other cardiovascular diseases. In insulin deficiency or insensitivity, hormone‐sensitive lipase is activated, leading to increased mobilization of free fatty acids (FFAs) from the peripheral depots with decreased peripheral utilization. The excess FFAs are catabolized to acetyl‐CoA. The acetyl‐CoA cannot be readily utilized as the availability of oxaloacetate is reduced and tricarboxylic acid (TCA) cycle activity is sluggish. This gives rise to the channeling of excess acetyl‐CoA to the pathway of cholesterol biosynthesis, leading to hypercholesterolemia and hyperlipidemia. These conditions are characterized by elevated levels of cholesterol, triacylglycerol, and low‐density lipoprotein (LDL) cholesterol (Muruganandan, Srinivasan, Gupta, Gupta, & Lal, 2005).

Glycemic index is a classification of the blood glucose‐raising potential of carbohydrate foods relative to glucose or white bread. Generally, there are three categories of foods based on their GI values: The high‐GI foods (≥70), intermediate‐GI foods (>55–≤70), and low‐GI foods (≤55) (Dona, Guilhem, Robert, & Philip, 2010; Eleazu, 2016).

The mainstay of management of diabetes mellitus is pharmacological (exogenous insulin or hypoglycemic drugs administration) or nonpharmacological (diet, exercise, and surgery) treatments (Achi, Ohaeri, Ijeh, & Eleazu, 2017). Dietary therapy, though not alleviating the necessity for insulin therapy, is designed to enhance insulin therapy and maintain glycemic regulation by helping in minimizing postprandial fluctuations in blood glucose (Nelson, 2008).

In Nigeria, medical practitioners, healthcare providers, and dieticians who are knowledgeable and skillful in the management and prevention of diabetes often recommend breadfruit (*Treculia africana*) as one of the foods that could be eaten by people with type 2 diabetes.

Although the study by Ajiboye et al. (2016) on *T. africana* fruit/seed suggested its beneficial use in the management of diabetes mellitus, our study (Eleazu et al., 2017) on processed *T. africana* seed gave contrary results, although the duration of our study was short. Furthermore, information on the effect of consumption of processed *T. africana* seed on the lipid profile of humans or animals is missing in literature. This is important as breadfruit is considered to be an oily seed which could impact on the lipid profile of humans or animals when consumed. In a bid to gain insight into the relevance of *T. africana* in glycemic control (using an extended study) and the impact its consumption will have on lipid profile of humans or animals, the present study was therefore designed with the following objectives:

1. To determine the effect of ethanol extracts (100 and 200 mg/kg) of *T. africana* seed on the oral glucose tolerance of normoglycemic rats.
2. To determine the effect of the extracts on the lipid profile (total cholesterol, low‐density lipoprotein [LDL] cholesterol, high‐density lipoprotein [HDL] cholesterol, and triacylglycerol) and body weights of normoglycemic rats.

## 2 MATERIALS AND METHODS

### 2.1 Plant materials

Dehulled seeds of *T. africana* were purchased from the meat market in Abakaliki, Ebonyi State, Nigeria. They were washed and boiled to softness, air‐dried to constant weight, and processed to flour using an electric blender.

### 2.2 Extraction of plant materials

The powdered seeds (903 g) were extracted with 2.5 L of absolute ethanol overnight in a big stoppered bottle with occasional stirring at room temperature. They were then sieved using muslin cloth. The filtrate was air‐dried for 24 hr to get the crude (ethanol) extract which was then reconstituted in normal saline. The percentage yield of the extract was calculated using this formula.

$$\%\text{yield}=\frac{\text{Weight of extract}}{\text{Weight of sample}}\times \frac{100}{1}$$

### 2.3 Experimental animals

Fifteen male albino rats of the Wistar strain (about 6 weeks old) obtained from a local farmer in Abakaliki were used for this study. Ethical approval was obtained from the Board of Department of Biochemistry, Ebonyi State University, Abakaliki, Nigeria, which was in line with the guidelines for the care and use of laboratory animals (NRC, 1985). The rats were kept in the animal house of the Department of Biochemistry, Ebonyi State University, Abakaliki. The animals were acclimatized for 2 weeks to their feeds and water which they had access to ad libitum. All the ethics for animal experiments were observed.

### 2.4 Experimental design

After acclimatization of the rats, they were randomly distributed into three groups of five rats per group as follows:

Group 1 received ethanol extract of *T. africana* seed at 100 mg/kg (Group 1).
Group 2 received ethanol extract of *T. africana* seed at 200 mg/kg (Group 2).
Group 3 (control group) received standard rat feeds and 1 ml/kg of normal saline in place of the extract. The extracts were administered daily throughout the duration of the experiment. The body weights of the rats were recorded on a daily basis using an electronic weighing balance.

### 2.5 Oral glucose tolerance test

Oral glucose tolerance tests were carried out just before administration of the extracts (at week 0) and after 2 and 4 weeks of administration of the extracts following the methods described by Chaturvedi, Georg, Milinganyo, and Tripathi (2004) and Taiwo, Odeigah, and Ogunkanmi (2009), respectively. To perform OGTT (at week 0), the rats in the respective groups were fasted overnight and, the next day, blood samples were collected from the tail vein of each rat just before (0 min) glucose administration at 30, 60, and 120 min, respectively, after glucose administration (3 g/kg) and their glucose levels were immediately estimated using a glucometer.

To perform OGTT (at weeks 2 and 4), rats were fasted overnight and, the next day, blood samples were collected from the tail vein of each rat just before (0 min) glucose administration at 15, 30, 45, 60, 90, and 120 min, respectively, after glucose administration (3 g/kg) and their glucose levels were immediately estimated using a glucometer. From the data generated from the OGTT at weeks 2 and 4, the positive incremental area under the curve (IAUC) was calculated for each group using the equation:

$$\begin{array}{cc} \text{IAUC}= & \left[(t1-t0)÷2]\times (C0+C1\right)+\left[(t2-t1)÷2]\times (C1+C2\right)\\ & +t\left[(t3-t2)÷2]\times (C3+C2\right)\ldots \end{array}$$

(where *t* = time and C = concentration of glucose) (Brouns et al., 2005; Olorunfemi, Patrick, Eyong, & Oladoja, 2010). Glycemic index was calculated as follows:

$$\text{GI}=\frac{\text{[IAUC test]}}{\text{[IAUC control]}}\times 100$$

At the end of 28 days, the rats were fasted overnight and euthanized on the 29th day by cervical dislocation. Blood was collected by cardiac puncture into plain tubes and allowed to clot at room temperature. Sera were harvested from the clotted blood samples following centrifugation at 4,000 *g* for 5 min for the analysis of lipid profile.

The percentage change in body weight of the rats was calculated as follows:

$$\text{Percentage change in weight}=\frac{\text{Final weight ‐ Initial weight}}{\text{Final weight}}\times 100$$

### 2.6 Lipid profile determination

The serum levels of total triacylglycerols, total cholesterol, and HDL cholesterol in the rats were analyzed using the Randox assay diagnostic kits following the methods described by Tietz (2005) and NCEP (2001). The LDL cholesterol and VLDL cholesterol levels were calculated using the method of Friedewald, Levy, and Fredrickson (1972) as described below:

$$\text{VLDL}=\frac{\text{Total triacylglycerol}}{5}$$

$$\begin{array}{cc} \text{LDL cholesterol}= & \text{Total cholesterol}-\text{(HDL cholesterol}\\ & \text{+}\text{VLDL cholesterol)} \end{array}$$

### 2.7 Calculation of atherogenic and coronary risk indices

The atherogenic index of the rats was calculated using the formula shown below as described by Omonkhua, Onoagbe, Ajileye, Aladegboye, and Adetoboye (2013) as follows:

Atherogenic index=LDL cholesterol−HDL cholesterol

The coronary risk index of the rats was calculated using method of Abbott, Wilson, Kannel, and Casteli (1998) and of Alladi and Shanmugasundaram (1989) as follows:

$$\text{Coronary risk index}=\frac{\text{Total cholesterol}}{\text{HDL cholesterol}}$$

### 2.8 Statistical analysis

Data were analyzed statistically using the Statistical Package for Social Sciences version 20.00. The comparison of means between the groups was made using one‐way analysis of variance (ANOVA) followed by Tukey's range test. The limit of statistical significance was set at *p* < .05.

## 3 RESULTS

**Figure 1:**
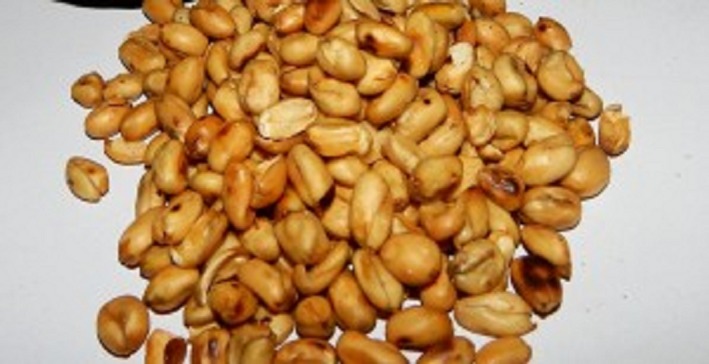
Pictorial representation of *Treculia africana* seeds

The pictorial representation of *T. africana* seeds that were used for this study is shown in Figure 1.

The percentage yield of the extract was obtained as 12.5%. The breadfruit seeds used for this study had earlier been shown to contain 1.64% ash, 18.58% crude protein, 1.33% fat, 1.33% crude fiber, 78.53% carbohydrate, 5.42 mg/100 g calcium, 0.83% phenols, and 1.32% flavonoids (at 10% moisture content) (Ijeh, Ejike, Nkwonta, & Njoku, 2010). The standard rat feeds that were used for the study contained 15% crude protein, 7% fat, 10% crude fiber, and 1% calcium.

**Figure 2:**
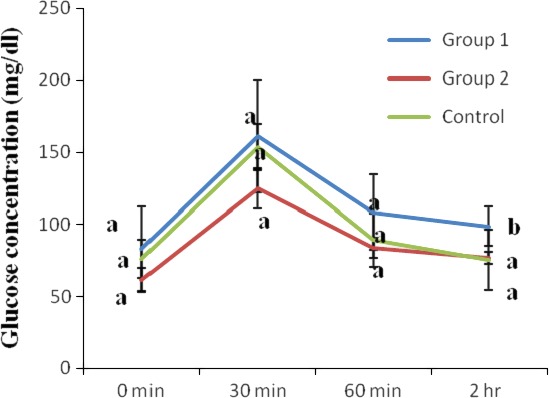
Oral glucose tolerance of glucose‐loaded normal rats (before extract administration). Values are reported as means ± *SD* (*n* = 5). a–b Means with different superscripts are significantly different for each group (*p* < .05)

The results of the oral glucose tolerance of glucose‐loaded normal rats carried out at week 0 (before administration of *T. africana* seed extract) are shown in Figure 2. As shown in the Figure, the blood glucose of the control group peaked to 153.33 mg/dl at 30 min post oral glucose loading but declined to 75.40 mg/dl after another 90 min. The blood glucose of group 1 rats peaked to 161 mg/dl at 30 min post oral glucose loading but declined to 98.75 mg/dl after another 90 min. The blood glucose of group 2 rats peaked to 125 mg/dl at 30 min postoral glucose loading but declined to 76.60 mg/dl after another 90 min.

**Figure 3:**
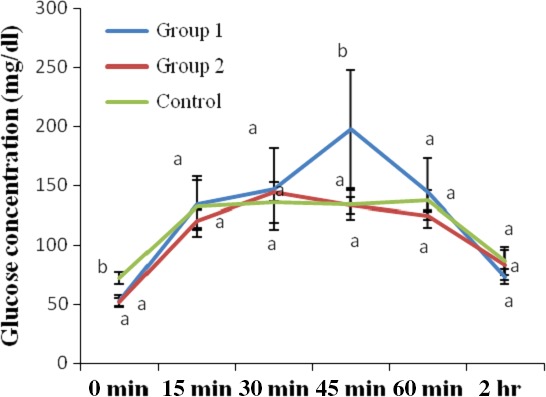
Oral glucose tolerance of glucose‐loaded normal rats after 2 weeks of administration of ethanol extract of *Treculia africana*. Values are reported as means ± *SD* (*n* = 5). a–b Means with different superscripts are significantly different for each group (*p* < .05)

The results of the OGTT of the normoglycemic rats after 2 weeks of administration of ethanol seed extract of *T. africana* seeds are shown in Figure 3. As shown in the Figure, the blood glucose of the control group peaked to 135.60 mg/dl at 30 min post oral glucose loading but declined to 86.40 mg/dl after another 90 min. The blood glucose of group 1 rats (administered *T. africana* at 100 mg/kg), peaked to 197.40 mg/dl at 45 min post oral glucose loading but declined to 73.20 mg/dl after further 75 min. The blood glucose of group 2 rats (administered *T. africana* at 200 mg/kg), peaked to 144.50 mg/dl at 30 min post oral glucose loading but declined to 83 mg/dl after further 90 min. When comparisons of OGTT were made between weeks, it was observed that at week 2 in comparison with week 0, administration of the *T. africana* seed extracts at 100 mg/kg to the rats of group 1, resulted in 57.58%, 9.52%, and 34.9% decreases of their blood glucose levels at 0, 30, and 120 min, respectively, but 24.65% increase in blood glucose at 60 min post oral glucose loading. In addition, administration of the *T. africana* seed extracts at 200 mg/kg to the rats of group 2 resulted in 19.61% and 13.49% decreases of their blood glucose levels at 0 and 30 min, respectively, but 32.85% and 7.71% increase in blood glucose at 60 and 120 min post oral glucose loading.

**Figure 4:**
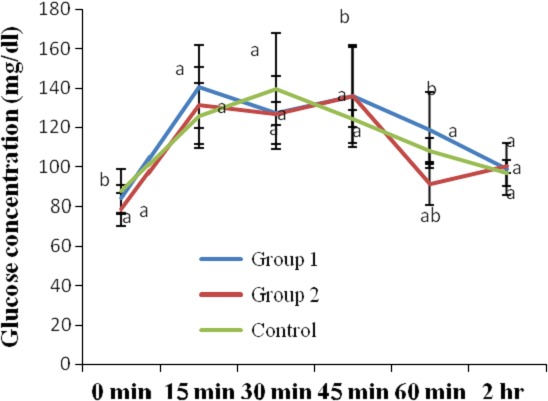
Oral glucose tolerance of glucose‐loaded normal rats after 4 weeks of administration of ethanol extract of *Treculia africana*. Values are reported as means ± *SD*. a–b Means with different superscripts are significantly different for each group (*p* < .05)

The results of the OGTT of the rats administered ethanol extracts of *T. africana* for 4 weeks are shown in Figure 4. As shown in the Figure, the blood glucose of the control group peaked to 139.6 mg/dl at 30 min post oral glucose loading but declined to 96.60 mg/dl after another 90 min. The blood glucose of group 1 rats (administered *T. africana* at 100 mg/kg for 4 weeks), peaked to 136.2 mg/dl at 45 min post oral glucose loading but declined to 98.60 mg/dl after further 75 min. The blood glucose of group 2 rats (administered *T. africana* at 200 mg/kg for 4 weeks), peaked to 135.80 mg/dl at 45 min post oral glucose loading but declined to 100 mg/dl after further 75 min. At week 4 with respect to week 2, administration of the *T. africana* seed extracts at 100 mg/kg to the rats of group 1 resulted in 36.99%, 4.41%, and 25.76% increases of their blood glucose levels at 0, 15, and 120 min, respectively, but 15.38, 44.93%, and 21.42% decreases in blood glucose at 30, 45, and 60 min post oral glucose loading. Furthermore, administration of the *T. africana* seed extracts at 200 mg/kg to the rats of group 2 resulted in 34.31%, 7.63%, 2.06%, and 17% increases of their blood glucose levels at 0, 15, 45, and 120 min, respectively, but 13.96% and 36.81% decreases in blood glucose at 30 and 60 min post oral glucose loading.

The IAUC and glycemic indices of the ethanol extracts of *T. africana* seed extracts are shown in Table 1. As shown in the Table, the IAUC for the standard rat feeds (given to the control group) *T. africana* seed extract at 100 mg/kg (administered to group 1 rats) and *T. africana* seed extract at 200 mg/kg (administered to group 2 rats) by the second week of experimentation were 7,594.5, 8,658, and 7,297.5, respectively. By the fourth week of experimentation, the IAUC for the standard rat feeds *T. africana* at 100 and 200 mg/kg were obtained as 7,315.5, 7,581, and 7,174.5, respectively. The glycemic indices for the standard rat feeds and *T. africana* seed extracts at 100 and 200 mg/kg by the second week of experimentation as calculated from their IAUC values were obtained as 100, 114, and 96.09, respectively. However, by the fourth week of experimentation, the GI values for the *T. africana* seed extract at 100 mg/kg decreased to 103.63 (10.0% decrease), while the GI values for the *T. africana* seed extract at 200 mg/kg increased to 98.07 (2.02% increase) as compared to the control group with a GI of 100.

**Table 1:** Incremental area under the OGTT curve (IAUC) and glycemic indices of the breadfruit extracts

Groups	Week 0 IAUC	Week 2 IAUC	Week 4 IAUC	Week 0 IAUC	Week 2 IAUC	Week 4 IAUC
Control	ND	7,594.5	7,315.5	ND	100	100.00
Group 1	ND	8,658	7,581	ND	114	103.63
Group 1	ND	7,297.5	7,174.5	ND	96.09	98.07

GI, glycemic index; IAUC, incremental area under the curve; ND, not determined.

The results of the body weights and percentage change in the body weights of the rats are shown in Table 2. As shown in the Table, the body weights of groups 1 and 2 rats were significantly lower (*p* < .05) than those of the control group at week 0. However, by the last week of experimentation, the body weights of group 1 (17.89% increase) and group 2 rats (18.38% increase) were not significantly different (*p* > .05) from those of the control group that recorded 12.38% increase in body weights.

**Table 2:** Body weights (g) and percentage change in the body weights of rats

Groups	Week 0	Week 2	Week 4	Percentage change
Control	151.06 ± 16.99^b^	164.46 ± 25.77^b^	172.40 ± 20.97^a^	12.38 (increase)
Group 1	124.80 ± 19.10^a^	149.60 ± 28.34^ab^	152.00 ± 27.22^a^	17.89 (increase)
Group 2	123.08 ± 17.23^a^	114.96 ± 41.76^a^	150.80 ± 20.433^a^	18.38 (increase)

Values are expressed as means ± *SD*.

Means with different superscripts (a–b) along each column are significantly different (*p* < .05).

**Figure 5:**
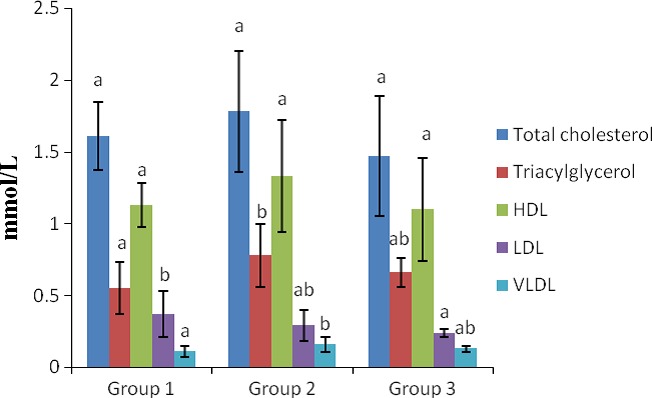
Lipid profile in the sera of rats. a–b Means with different superscripts are significantly different for each group (*p* < .05). Groups 1, 2, and 3 represent the study groups 1 and 2 and the control groups, respectively

The results of the serum lipid profiles of the rats are shown in Figure 5. As shown in the Figure, the total cholesterol levels of the group 1 rats were not significantly different (*p* > .05) from those of the control group. Similarly, the total cholesterol levels of group 2 rats were also not significantly different (*p* > .05) from those of the control group.

The serum total triacylglycerol levels of the group 1 rats were not significantly different (*p* > .05) from those of the normal control group. Furthermore, the total triacylglycerol levels of the group 2 rats were also not significantly different (*p* > .05) from those of the control group.

The HDL cholesterol levels of the group 1 rats were not significantly different (*p* > .05) from those of the control group. In addition, the HDL cholesterol levels of the group 2 rats were also not significantly different (*p* > .05) from those of the control group.

The LDL cholesterol levels of the group 1 rats were significantly increased (*p* < .05) when compared with the control group. Administration of the *T. africana* extract at 200 mg/kg to the rats resulted in nonsignificant elevation (*p* > .05) of their LDL cholesterol levels when compared with the control group.

The VLDL cholesterol levels of the group 1 rats were not significantly different (*p* > .05) from those of the control group. Following administration of the *T. africana* extract at 200 mg/kg to the rats of group 2, the values obtained for their VLDL cholesterol levels were not significantly different (*p* > .05) from those of the control group.

**Figure 6:**
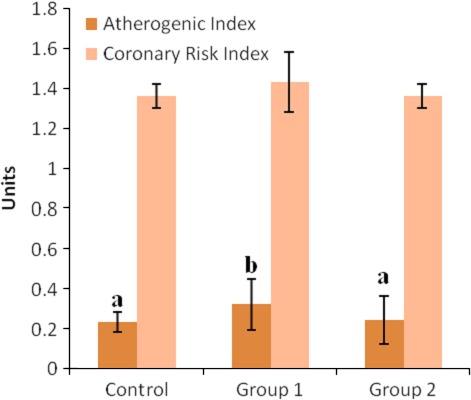
Atherogenic and coronary risk indices of rats. a–b Means with different superscript letters are significantly different across the groups (*p* < .05). Means without superscript letters are not significantly different across the groups (*p* > .05). Groups 1, 2, and 3 represent the study groups 1 and 2 and the control groups, respectively

The results of the atherogenic and coronary risk indices of the rats are shown in Figure 6. The atherogenic and coronary risk indices of the group 1 rats were significantly increased (*p* < .05) when compared with the control group. On the contrary, the atherogenic and coronary risk indices of the group 2 rats were not significantly different (*p* > .05) from those of the control group.

## 4 DISCUSSION

The OGTT is a good and cheap measure of insulin secretion, sensitivity, and glucose uptake (Rhee et al., 2010). The test measures an individual's ability to utilize ingested glucose over a given period of time (Achi et al., 2017; Eleazu et al., 2017; Olorunfemi et al., 2010).

For normal tolerance to blood glucose, blood glucose concentration at 2 hr following oral glucose loading is about 110 mg/dl, whereas for impaired tolerance, blood glucose rises above 110 mg/dl due to lack or insensitivity of insulin, resulting in reduced glucose uptake and its utilization by the muscle and adipose tissues. As impaired oral glucose tolerance indicates the predisposition of an organism to diabetes, medicinal plants that possess antidiabetic actions will improve glucose tolerance, thereby stopping the progression of impaired glucose tolerance to diabetes (Achi et al., 2017; Shivananda & Shivananda, 2007). Therefore, the findings of this study which indicated that the oral glucose tolerance levels of groups 1 and 2 rats administered the ethanol extracts of *T. africana* (at 100 and 200 mg/kg) for 2 weeks declined to values ≤100 mg/dl suggest normal tolerance to glucose following administration of the extracts at the doses used in this study. However, when comparisons of OGTT were made within the weeks, it was observed that at week 2 in comparison with week 0, administration of the *T. africana* seed extracts at 200 mg/kg to the rats of group 2 stimulated increased glycemia at 60 and 120 min post oral glucose loading. Furthermore, when the blood glucose levels of the rats of groups 1 and 2 at week 4 were compared with their blood glucose levels at week 2, it was observed that administration of the *T. africana* seed extracts at 100 and 200 mg/kg to the rats of groups 1 and 2, respectively, stimulated increased glycemia at 120 min. These findings suggest that boiling of *T. africana* seed impacts it with a high glycemic index. In our previous study (Eleazu et al., 2017), we reported that dietary intake of boiled *T. africana* seeds did not improve hyperglycemia in streptozotocin‐induced diabetic rats. Therefore, findings of this study suggests that the normal glucose tolerance in the groups 1 and 2 rats following oral glucose loading could be a result of the beta cells of the pancreas of the animals being able to release insulin, enhancing uptake of glucose in the insulin‐responsive tissues and not necessarily as a result of the extracts they were administered. This is further buttressed by the inability of this plant to ameliorate the impaired glucose tolerance of streptozotocin‐induced diabetic rats as previously reported (Eleazu et al., 2017) and by the observed normal glucose tolerance in the control group following oral glucose loading. This finding leads us to suggest that consumption of *T. africana* by a nondiabetic subject may have no effect on the glucose tolerance of the individual, while it will negatively impact on the glycemic status of a diabetic subject.

To further explore the effect of *T. africana* seed on blood glucose, the GI of the *T. africana* seeds was determined.

Glycemic index is a classification of the blood glucose‐raising potential of carbohydrate foods relative to glucose or white bread (Wolever et al., 1994). Long‐term consumption of high‐GI foods was proposed to increase insulin demand, promote insulin resistance, impair pancreatic β‐cell function, and eventually lead to type 2 diabetes (Brand‐Miller, 2003). Therefore, the glycemic index values obtained for the ethanol extracts of the *T. africana* seeds at the doses that were used in this study further suggest that boiling of *T. africana* seed impacts it with a high glycemic index.

The increase in the body weights of the groups 1 and 2 rats at the last week of experimentation when compared with the control group could be attributed to the rich amount of amino acids in *T. africana* seeds and their high biological value that even exceeds that of soybean (Adesina & Adeyeye, 2016).

Hyperlipidemia is a lipid disorder that alters lipid profile. It is characterized by elevated serum total cholesterol, low‐density lipoprotein cholesterol, and very‐low‐density lipoprotein cholesterol, and decreased high‐density lipoprotein cholesterol levels though may be asymptomatic (Adejor, Danladi, Dorcas, Olumuyiwa, & Uche, 2016).

The nonsignificant differences in the serum levels of total cholesterol, triacylglycerol, HDL cholesterol, and VLDL cholesterol levels of the groups 1 and 2 rats compared with the control group and the nonsignificant difference in the LDL cholesterol levels of the group 2 rats compared with the control group suggest the nonhyperlipidemic action of *T. africana*. However, it was observed that the group 1 rats administered *T. africana* extracts at 100 mg/kg had increased levels of LDL cholesterol, a finding that corroborates previous reports of Okwari, Ofem, Ettarh, and Eyong (2006) on the LDL cholesterol upregulating action of *T. africana* seeds in alloxan‐induced diabetic rats. LDL cholesterol particles are referred to as bad cholesterol because they can transport their contents of lipid molecules into arterial walls attract macrophages, all of which result in atherosclerosis. Thus, increasing concentrations of LDL cholesterol particles are strongly associated with increasing rates of accumulation of atherosclerosis within the walls of arteries which over time, eventually results in sudden plaque ruptures, triggering clots within the artery opening (Dashty et al., 2014). The findings of this study with respect to the LDL cholesterol‐raising properties of boiled *T. africana* seed therefore call for caution in the consumption of this plant as a regular meal.

Atherogenic index expressed as the ratio LDL cholesterol to HDL cholesterol (Omonkhua et al., 2013) has been found to be a good predictor of atherosclerosis and cardiovascular disease risk as it is considered more prognostic than LDL cholesterol or HDL cholesterol alone (Dobiásová, 2006; Milada, 2004; Nadeem, Muhammad, & Yousaf, 2013). Therefore, the increased atherogenic index of the group 1 rats, as seen in this study, further affirms the atherogenic properties of boiled *T. africana* seed.

Coronary risk index is determined as the ratio of total cholesterol to HDL cholesterol (Omonkhua et al., 2013), and it is established by elevated levels of total cholesterol, especially LDL cholesterol (Temme, VanHoydonck, Schouten, & Kesteloot, 2002; Bagri, Ali, & Aderi, 2009). The increased coronary risk indices of the groups 1 rats suggest that consumption of this plant could predispose an individual to cardiovascular diseases. As *T. africana* seed is considered to be an oily plant, the study underscores the need to characterize the plant to determine the types of lipids in it.

## 5 CONCLUSION

The study showed that consumption of ethanol extract of boiled *T. africana* seed increased their body weights. Furthermore, the extract at 100 mg/kg increased their LDL cholesterol, AI, and CRI, suggesting that consumption of boiled African breadfruit may expose an individual to the risk of development of cardiovascular diseases. Finally, the study suggested that consumption of *T. africana* seed by a nondiabetic subject may have no effect on the glucose tolerance of the individual, while it will negatively impact on the glycemic status of a diabetic subject.

## CONFLICT OF INTEREST

None declared.
